# Taylor Swift does not boost face recognition in reaction time-based Concealed Information Test: investigating target-familiarity effects

**DOI:** 10.1007/s00426-024-02003-1

**Published:** 2024-09-04

**Authors:** Laure Z. Kohn Lukic, Nele Möck, Bruno Verschuere, Melanie Sauerland

**Affiliations:** 1 Department of Clinical Psychological Science, Faculty of Psychology and Neuroscience, Maastricht University, Maastricht, The Netherlands; 2 Department of Clinical Psychology, University of Amsterdam, Amsterdam, The Netherlands

## Abstract

Eyewitness identifications from lineups are prone to error. More indirect identification procedures, such as the reaction-time based Concealed Information Test (RT-CIT) could be a viable alternative to lineups. The RT-CIT uses response times to assess facial familiarity. Theory and initial evidence with autobiographical stimuli suggests that the accuracy of RT-CIT can be augmented when participants’ reliance on familiarity-based responding increases. We tested this assumption in two pre-registered experiments with 173 participants. Participants witnessed a mock crime. In the subsequent RT-CIT protocol, participants reacted to probe faces from the mock crime video, to irrelevant faces, and to target faces that required a unique response. Targets were either unknown people or were well-known celebrities (e.g., Taylor Swift). As expected, reaction times were longer to probes than to irrelevants in all conditions, indicating a CIT effect. Contrasting our pre-registered predictions, the CIT effect was not larger in the familiar condition (Experiment 1: unfamiliar targets: *d* = 0.77 vs. celebrity targets: *d* = 0.24; Experiment 2: unfamiliar targets: *d* = 1.09 vs. celebrity targets: *d* = 0.79). This suggests that familiar targets did not increase the validity of the RT-CIT in diagnosing concealed face recognition. A potential lack of saliency of the familiar targets might explain these unexpected findings. Of note, we did find medium to large effect sizes overall, speaking to the potential of diagnosing face recognition with the RT-CIT.

In 2010, a 28-year-old man was stabbed outside a nightclub in Houston, Texas. Several people reported seeing the attack and one of the witnesses called the authorities the next day claiming he spotted someone who looked like the killer. This led the police to suspect Lydell Grant. Several eyewitnesses viewed a photo lineup and six of them picked Grant as the perpetrator. Grant was convicted for first-degree murder and sentenced to life in prison. Eight years later, DNA evidence revealed that Grant was not the true perpetrator, and he was released from prison. Later it became clear that the lineup procedures were heavily biased (Possley, 2021). For example, the lineups were not conducted in a double-blind fashion (Wells et al., 2020) and witnesses received confirming feedback (see Douglass & Steblay, 2006, for a meta-analysis) after their lineup decisions (https://innocencetexas.org).

This case is not an exception. According to the Innocence Project (https://innocenceproject.org), 69% of 375 wrongful convictions they helped overturn were due to mistaken eyewitness identifications. Similarly, research suggests that identification errors in traditional lineup procedures are common (Fitzgerald & Price, 2015). New and more accurate identification procedures would therefore greatly assist police investigation and criminal justice. The Concealed Information Test (CIT; Lykken, 1959) might be such an alternative as it provides a more indirect assessment of recognition. It is the aim of the current paper to investigate its usefulness as an eyewitness identification procedure.

The Concealed Information Test was developed as an alternative to the traditional polygraph technique and aims to assess recognition memory rather than deception (for a comprehensive review see Verschuere et al., 2011). The capacity of the CIT for detecting recognition has been established in different memory paradigms involving a mock crime or autobiographical information about the participants and using physiological measures such as heart rate and skin conductance response as outcomes. Effect sizes for detecting recognition are large and vary between *d* = 0.89 and 1.89 for different physiological measures (Meijer et al., 2014).

The simplicity in administration has brought the focus to the *reaction time-based* RT-CITs in recent years (see Suchotzki et al., 2017, for a meta-analysis). The RT-CIT comprises three types of stimuli: irrelevant (control) stimuli, targets that warrant a unique response and assure attention to the stimuli, and probes for which the test procedure aims to assess recognition. Figure 1 shows an illustration of the different items used in different types of Concealed Information Tests. For example, participants view a detail of the crime that only the real perpetrator would know (e.g., the murder weapon). This is considered the probe (e.g., a baseball bat). Additionally, participants view several plausible alternatives (e.g., a pistol, a hammer, an axe). These stimuli are the irrelevants. An innocent person without crime knowledge would not be able to differentiate between the probe and the irrelevants. However, the real perpetrator would be expected to recognize the murder weapon, the probe, and should therefore take longer to respond to it. The third stimulus type is the target. Targets are similar to irrelevants in that they are plausible alternatives to the probe with the difference that participants become familiar with and memorize the targets just before the test. Additionally, participants receive instructions to respond with ‘Yes’ or ‘Recognized’ to targets, but with ‘No’ or ‘Not recognized’ to irrelevants and probes. The use of a response deadline prevents strategic slowing (Suchotzki et al., 2021).

**Fig. 1:**
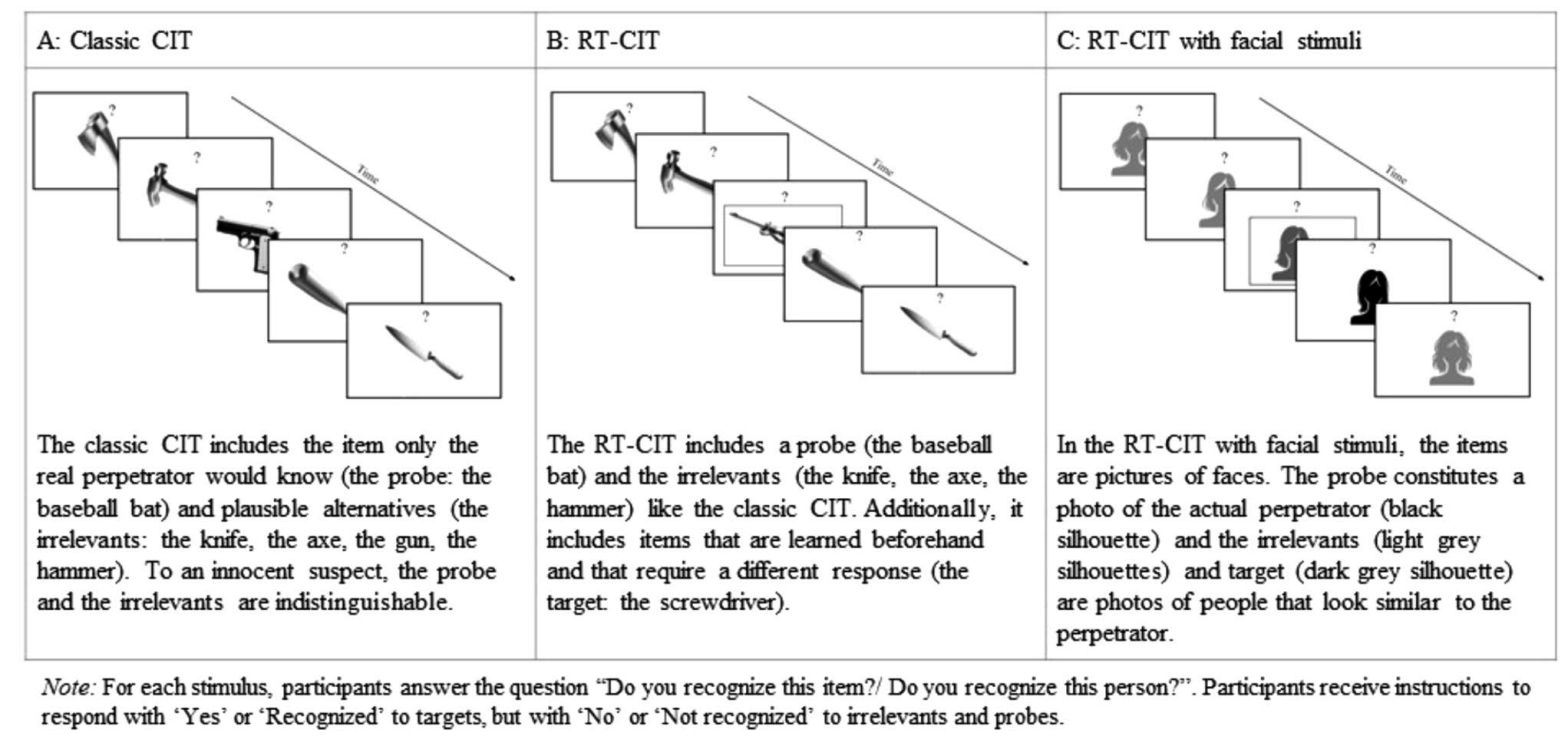
Illustration of classic CIT, reaction time-based CIT, and reaction time-based CIT with facial stimuli

Thirteen experiments thus far have applied RT-CIT for establishing face recognition (cf. panel C of Fig. 1). In a series of five experiments, participants watched a mock crime video depicting a theft (Experiment 1–4) or a virtual reality event showing the vandalism of a car (Experiment 5; Sauerland et al., 2019). Similar to the construction of a lineup, where the fillers match the description of the perpetrator and appearance of the suspect (Wells et al., 2020), the irrelevants and targets fitted the general description of the probes, the actors in the stimulus event. A comparison of the reaction times in response to the irrelevants and probes revealed a very small average effect size across the five experiments. Follow-up experiments decreased the number of protagonists in the mock crimes from four to two and have consistently reported moderate and sometimes large effect sizes (Georgiadou et al., 2022; Sauerland et al., 2023a, b; Sauerland et al., 2024).

One way to increase the validity of the CIT is to ensure that participants categorize the stimuli according to familiarity (Suchotzki et al., 2018). It is a typical finding in the memory literature that feelings of familiarity evolve fast and automatically (Yonelinas, 2002; Yonelinas et al., 2010). The RT-CIT relies on those fast and automatic responses to familiar stimuli (Verschuere & De Houwer, 2011). Familiarity with the probes evokes a ‘Yes’/’Recognized’ response, yet the task instructions require the examinee to answer ‘No’. This leads to a response conflict that delays the response times. Thus, the greater the reliance on familiarity, the greater the RT-CIT effect.

There are different ways of increasing reliance on familiarity during the CIT. For example, we can add familiarity-related fillers. Familiarity-related fillers are words that are semantically related to the concept of familiarity (e.g., ‘RECOGNIZED’, ‘MINE’, ‘UNKNOWN’, ‘FOREIGN’). In one experiment, these familiarity-related fillers appeared on the screen during the CIT protocol and participants had to categorize them as familiar or unfamiliar, similar to the target discrimination task (Lukács et al., 2017). As expected, this increased the RT-CIT effect (*d* = 1.54). Another way to enhance familiarity-based categorization is by including familiar targets^1^. For example, in a test of participants’ country of origin and birth date (probes), familiar targets were countries and dates that were familiar to participants, whereas unfamiliar targets were randomly chosen dates and countries (Suchotzki et al., 2018; but see Koller et al., 2024). In a first study, the CIT effect was larger in the familiar than the unfamiliar target condition and a second pre registered study confirmed these exploratory findings (familiar target condition: *d* = 1.79; unfamiliar target condition: *d* = 1.03).

Based on the beneficial value of familiar stimuli in CIT protocols, we tested the usefulness of familiar targets for increasing the CIT effect with facial stimuli in two pre-registered experiments. Participants watched a mock crime and completed an RT-CIT protocol. In the unfamiliar target condition, the targets were randomly picked from the irrelevant items and participants learned them before starting the CIT protocol. In the familiar target condition, the targets were well-known celebrities whose general appearance matched the protagonists in the mock crime video, namely Selena Gomez, Katy Perry, Taylor Swift, and Emma Watson. These targets were also memorized before starting the CIT. Because the celebrity stimuli differed in several aspects compared to the unfamiliar targets (e.g., lighting, amount of make-up) they probably stood out against the other stimuli in Experiment 1 (see also Kubo & Nittono, 2008). In Experiment 2, we therefore edited the familiar targets to look less distinctive. We expected that the recognition of the protagonists in the stimulus film (probes) would be reflected in longer reaction times, compared to reaction times of irrelevants (RT-CIT effect, hypothesis 1). Second, we expected the RT-CIT effect to be moderated by target-familiarity (unfamiliar vs. familiar targets, hypothesis 2). Thus, we expected a larger CIT effect in the familiar condition compared to the unfamiliar condition.

## Methods

We pre-registered both experiments on the open science framework (Experiment 1: https://osf.io/7ny5g; Experiment 2: https://osf.io/zdu5x. The Ethics Review Committee of the Faculty approved both experiments (approval codes: OZL_231_140_12_2020_S7 and 231_140_12_2020_S8). The Inquisit scripts, pilot data, and data are available here: https://osf.io/j6vuf/?view_only=. We cannot share all stimuli because we do not have permission of the depicted individuals and due to copyright reasons (familiar targets).

### Deviations from pre-registration

There are several deviations from the pre-registration: first, in addition to the analyses across the two probes, we conducted separate analyses for the thief and the victim, in line with earlier work (Sauerland et al., 2019, 2023a). Second, we pre-registered Bayesian analyses for Experiment 1 but by oversight did not include it in the pre-registration for Experiment 2. We conducted the Bayesian analyses for both experiments for consistency.

### Participants

#### Power analyses

For Experiment 1, we conducted a power analysis using *MorePower 6.0.4* (Campbell & Thompson, 2012). We based the estimated effect size on Suchotzki et al. (2018; Experiment 2) who reported an interaction effect between stimulus type and target familiarity of η_p_^2^ = .25. We set the alpha error probability to .05, and power to .95. This led to a required sample size of 44 participants (22 per condition), or 52 participants (26 per condition) when accounting for dropouts and exclusions. However, because initial studies typically overestimate the true effect size (Camerer et al., 2018) and particularly interaction effects have been difficult to replicate, we aimed to test at least double this amount (i.e., *N* ≥ 104).

For Experiment 2, we conducted a power analysis with G*Power 3.1.9.7 (Faul et al., 2007). Based on the null results of Experiment 1, we considered a lower effect size (i.e., a medium effect size of η_p_^2^ = .06), set power to .95 and the α-error probability to .05. The power analysis returned a required sample size of 54 participants. Considering a likely dropout and exclusion rate of +/- 15% we aimed to test at least 64 participants or as many as we could within two months.^2^

#### Samples

In Experiment 1, we tested 109 participants. We excluded 12 participants, because they had an error rate of more than 50% (Kleinberg & Verschuere, 2015), four participants because they answered more than one attention check question wrong (Sauerland et al., 2023a, b), and three participants dropped out during the experiment. The final sample consisted of 90 participants (*M*_*age*_ = 23.41, *SD*_*age*_ = 3.88, range = 19–46). The unfamiliar-targets and the familiar-targets condition included 41 and 49 participants, respectively. Participants (75.6% female, 23.3% male, 1.1% non-binary) were bachelor students (51.1%), master students (45.6%), or indicated no university track (3.3%). Most participants were students at the Faculty of Psychology and Neuroscience (51.1%) or not a student at this University (36.7%). Participants’ most common native languages included German (31.1%), English (21.1%), Luxembourgish (14.4%), and Dutch (13.3%).

In Experiment 2, we tested 97 participants. Ten participants dropped out and we excluded four participants for one of the following reasons: an error rate of more than 50% in response to the targets, completing the study twice, recognizing one of the actresses in the stimulus film from real life, or failing two of the three attention check questions. The final sample consisted of *N* = 83 participants (*M*_*Age*_ = 21.4 years, *SD*_*Age*_ = 5.2, range = 17–54 years). Participants (75.9% females, 21.7% males, 2.4% non-binary) were mostly Bachelor students (90.4%) and Master students (4.8%). Most participants were students at the Faculty of Psychology and Neuroscience (86.7%) or not a student at this University (9.6%). The most common native languages were German (50.6%), Dutch (22.9%) and Greek (13.8%).

#### Design

We used a 2 (target familiarity: familiar vs. unfamiliar) x 2 (stimulus type: probe vs. irrelevants) mixed design and manipulated target familiarity (familiar vs. unfamiliar) between subjects and stimulus type (probe vs. irrelevants^3^) within subjects in both experiments.

### Materials

#### Stimulus films

As a base for the two stimulus films, we used the four films from Sauerland et al. (2023a). In these films, one of two protagonists was visible primarily from the front and one primarily from the side. For the current study, we cut the four films to receive two films that included frontal close-ups and some distant profile views of both protagonists. The two films show the same action with two women: on a square in a pedestrian zone, the future thief walks up to the future victim to ask for directions. In the next scene, the victim is sitting down and is busy on her phone. Sneaking up from behind, the thief steals the victim’s purse. The actors switched between roles of thief and victim in the two versions to avoid confounding effects due to the actor or role. Table 1 contains an overview of the facial viewing times of the protagonists in each stimulus film, split into close-ups and distant shots of the face in a frontal and profile view.

**Table 1:** Facial viewing times and observation times for two stimulus films in seconds (s)

Film version	Film version 1		Film version 2	
Role (Actor)	Thief (A)	Victim (B)	Thief (B)	Victim (A)
Close-up frontal view	14	24	15	21
Distant frontal view	3	2	5	5
Close-up profile View	2	2	2	8
Distant profile view	13	11	14	10
Overall facial exposure time	32	39	36	44
Overall film duration	69		69	

#### Facial stimuli

Fourteen photographs included the two probes, four targets, and eight irrelevants. Two targets and four irrelevants each matched the general description of one of the probes, as determined in earlier research (Sauerland et al., 2023a). The photographs showed faces from a frontal view from the collarbone up, with loose, open hair, without jewelry, accessories, or glasses and exhibited a neutral expression. All clothing was edited to be black.

In the unfamiliar target condition, we randomly selected two targets from the non-probe photographs per probe. In the familiar target condition, the targets consisted of four celebrities.

#### Celebrity faces

To select the celebrities, we conducted two pilot studies. Our aim was to identify four celebrities that matched the general description of the probes (cf. Sauerland et al., 2019; Wells et al., 2020) and were generally well-known in a student population. For the first pilot, we selected 12 celebrities that matched the general description of the two probes (six for each probe). Thirty participants (18 women, 9 men, 2 non-binary, 1 prefer not to say; *M*_age_ = 21.63, *SD*_age_ = 2.48) sequentially viewed the celebrity faces on Qualtrics and indicated whether they knew them. Participants could either indicate celebrities’ names or where they knew them from. More than 90% of the participants were familiar with Selena Gomez and Emma Watson. The other celebrities reached only 66% familiarity or less or were found too old to match the 21-year old probes in hindsight (i.e., Jennifer Aniston, Scarlet Johansson). We therefore conducted a second pilot with another set of 12 celebrities. From this pilot, we selected Taylor Swift and Katy Perry as familiar targets, based on familiarity scores of 93% and 83%, respectively. Thus, the four familiar targets were Selena Gomez, Katy Perry, Emma Watson, and Taylor Swift.

Once we had established the identity of the familiar targets, we searched the internet with the celebrities’ names as the search term on Google Images to obtain high quality photographs with an unobstructed facial frontal view. In Experiment 1, we edited the chosen images using Adobe Photoshop (2022) to match the appearance of the unfamiliar target photos. We adjusted the background to a solid white and cropped the photos from the shoulders be the same size as the unfamiliar stimuli. We edited out all jewelry and accessories and adjusted the clothes to black shirts.

In Experiment 2, we further edited the familiar targets to look less distinctive. For example, celebrities wore heavy make-up and lighting was distinctive to photographs taken in a regulated room. We adjusted the colors and levels using the *Camera Raw Filter* in Photoshop to match the color grading to the pictures. Additionally, we reduced make-up intensity. Finally, we reduced the picture quality by lowering the resolution and adding noise to match the lower quality of the other pictures.

#### Celebrity information (Experiment 2)

To improve familiar target recognition in Experiment 2, participants received information about and photos of each of the celebrities at the beginning of the testing session, prior to the CIT protocol. For example, participants saw three photos of Taylor Swift alongside her occupation, her most known songs and the number of Grammy awards she won.

#### Reaction time-based Concealed Information Test

The RT-CIT of was programmed using Inquisit 6.5.2 Lab (Experiment 1) and Inquisit 6.6.1 Lab (Experiment 2). The data were recorded in milliseconds, using one joint CIT protocol for the thief and victim. All photos were 388 × 462 pixels in size. We followed a similar protocol as other RT-CIT studies with facial stimuli (e.g., Georgiadou et al., 2022; Sauerland et al., 2023b). Participants received instructions to press the ‘L’ key on the keyboard as fast as possible in response to a facial stimulus, with the exception of the two targets. For these stimuli, they should press the ‘A’ key. We included the names of the celebrities in the instructions of the familiar targets condition. Participants then viewed the four targets for 30 s, accompanied by instructions to encode these faces. A practice block followed, showing each photo (2 × 2 targets, 2 × 4 irrelevants, and 2 × 1 probes) once. Above each facial stimuli, the question ‘Do you recognize this person?’ appeared. The labels ‘Yes’ and ‘No’ appeared to the left and right sides of each stimulus, respectively. Participants had 1500 ms to respond. After 800 ms, they received the warning ‘Too slow!’ Participants received the feedback ‘Wrong’ when they responded different than instructed. We randomly set the inter-stimulus interval between 250, 500, or 750 ms. A second, identical practice block followed if participants made more than one mistake or if they had responded too slow. Preceding the second practice block, participants received reminder instructions on how to respond. After this second practice block, participants continued to the actual task regardless of performance. Before the task started, the instructions appeared again, and participants viewed the targets for five more seconds. Similar to previous experiments, participants viewed the targets for a total of 35 s (Sauerland et al., 2023b). During the actual task, every stimulus appeared 18 times, at a random sequence. The labels and feedback were identical to the practice blocks. In total, the actual task consisted of 252 trials (2 × 7 × 18).

The RT-CIT of Experiment 2 was identical to the first experiment except that the participants had to complete three practice phases rather than one or two. During the first practice phase, the stimulus only disappeared once participants pressed the A or L key. This way, participants could control the pace of the test. They already received the ‘Wrong’ feedback if applicable. In the second practice phase, the stimuli disappeared after 1500 ms, if no key was pressed. The last practice trial was identical to the second practice trial except that participants additionally received feedback on their speed, with the words ‘Too slow!’ appearing on the screen after 800 ms. Participants viewed each of the 14 stimuli twice during each training phase. If a participant made too many mistakes during one of the practice phases, they had to repeat it once again. To make sure that the target photos were memorized, they were shown for five seconds and the instructions were repeated after each practice phase and before the actual task began.

#### Follow-up photo display

After completion of the RT-CIT task, participants viewed a photo recognition display that showed all 14 stimuli used, arranged in four rows, alternating between three and four photos. The 14 photographs included the two probes, four targets, and eight irrelevants. Two targets and four irrelevants each matched the general description of one of the probes, as determined in earlier research (Sauerland et al., 2023a). The images were numbered (1, 2, 3, etc.). Participants indicated which individuals they recognized from the video. This allowed us to roughly determine if participants in the CIT conditions had explicit memory of the probes.

#### Attention check

Participants answered three forced-choice attention check questions with four or five answer options. The forced-choice questions were ‘Where did the actors first meet?’; ‘What was the victim doing when the handbag was stolen?’, and ‘What color was the stolen handbag?’ We excluded participants if they did not answer at least two of the three questions correctly.

#### Procedure

Recruitment occurred online, using the University’s research participation system Sona, social media sites such as Facebook, Whatsapp and Instagram, and other research platforms (e.g., SurveyCircle, 2022). Participants received instructions to complete the study on a laptop or PC and that they may have to download a plug-in. Participants could not take part if they had participated in one of the pilot studies or previous RT-CIT experiments in the department. After reading an information letter and providing consent, the software randomly allocated participants to one of two conditions (familiar vs. unfamiliar). Participants received instructions to watch the film closely and to pay close attention because they would answer questions about the film later. After the film, participants answered the attention check questions and in Experiment 2, participants in the familiar condition additionally read the information about the celebrities. Subsequently, participants completed the RT-CIT task. Participants then indicated the probes in the follow-up photo display and indicated whether they recognized any of the individuals in the study except for the celebrities from ‘real life’. Finally, participants were debriefed and thanked for their participation. The studies lasted approximately 20 to 30 min. SONA participants received credit for their participation.

## Results

### Data preparation

Exclusion criteria in both experiments included answering less than 2 out of 3 attention checks correctly (Sauerland et al., 2023a, b), and displaying a high error rate or non-completed trials (i.e., ≥ 50%; cf. Kleinberg & Verschuere, 2015). Based on earlier RT-CIT experiments with facial stimuli, we only included correct trials with reaction times between 150 ms and 1500 ms (Georgiadou et al., 2022; Sauerland et al., 2023b). Correct trials consisted of pressing ‘A’ for targets and pressing ‘L’ for all other facial stimuli. We aggregated reaction times into mean reaction times per stimulus type (probes, targets, and irrelevants). In addition to the pre-registered analyses, we also present separate results for the thief and the victim, in addition to the analyses collapsed across both probes.

### Pre-registered analyses: RT-CIT effect and moderation by familiarity

Table 2 shows the mean reaction times and inferential statistics for the comparison of reaction times for probes and irrelevants for Experiment 1 and 2. We conducted a 2 (stimulus type: probe vs. irrelevants) x 2 (target familiarity: familiar vs. unfamiliar) mixed measures ANOVA to test the effect of familiarity on the CIT effect. For Experiment 1, the main effect of stimulus type was significant, *F*(1,88) = 22.73, *p* < .001, *n*_*p*_^*2*^ = .205, as expected. Supporting hypothesis 1, the reaction times for probes (*M* = 515 ms, *SD* = 70) were slower than for irrelevants (*M* = 502 ms, *SD* = 56), *d* = 0.47, 95% CI [0.25; 0.69], revealing a moderate CIT effect. Additionally, the main effect of familiarity was significant, *F*(1,88) = 51.99, *p* < .001, *n*_*p*_^*2*^ = .371. Specifically, reaction times were slower in the unfamiliar condition (*M*_*Probe*_ = 560 ms, *SD*_*Probe*_ = 61; *M*_*Irrelevant*_ = 538 ms, *SD*_*Irrelevant*_ = 43) than in the familiar condition (*M*_*Probe*_ = 478 ms, *SD*_*Probe*_ = 54; *M*_*Irrelevant*_ = 471 ms, *SD*_*Irrelevant*_ = 45).

**Table 2:** Descriptives and inferential statistics for pairwise comparisons of the reaction times (ms) for probes and irrelevant stimuli in Experiment 1 and 2

Target-familiarity	Role	df	*t*	*d*	*p*	Mean response time in ms (*SD*)	
						Probes	Irrelevants
Experiment 1							
Unfamiliar	Thief	40	4.50	0.70	≤ .001	560 (62)	536 (46)
	Victim	40	3.08	0.48	.004	565 (77)	541 (46)
	Across roles	40	4.94	0.77	≤ .001	560 (61)	538 (43)
Familiar	Thief	48	1.78	0.25	.081	478 (56)	470 (47)
	Victim	48	1.02	0.15	.314	478 (58)	472 (46)
	Across roles	48	1.66	0.24	.103	478 (54)	471 (45)
Across familiarity conditions	Thief	89	4.32	0.46	≤ .001	515 (71)	500 (57)
	Victim	89	2.98	0.31	.004	517 (80)	503 (57)
	Across roles	89	4.43	0.47	≤ .001	515 (70)	502 (56)
Experiment 2							
Unfamiliar	Thief	47	3.17	0.46	.001	544 (50)	527 (45)
	Victim	47	6.72	0.97	≤ .001	566 (49)	530 (42)
	Across roles	47	7.54	1.09	≤ .001	554 (41)	528 (42)
Familiar	Thief	34	4.23	0.72	≤ .001	485 (44)	468 (40)
	Victim	34	3.28	0.56	.001	487 (49)	471 (37)
	Across roles	34	4.65	0.79	≤ .001	486 (42)	469 (38)
Across familiarity conditions	Thief	82	4.83	0.53	≤ .001	519 (55)	502 (52)
	Victim	82	7.15	0.79	≤ .001	533 (63)	505 (50)
	Across roles	82	8.70	0.95	≤ .001	525 (53)	503 (49)

**Fig. 2:**
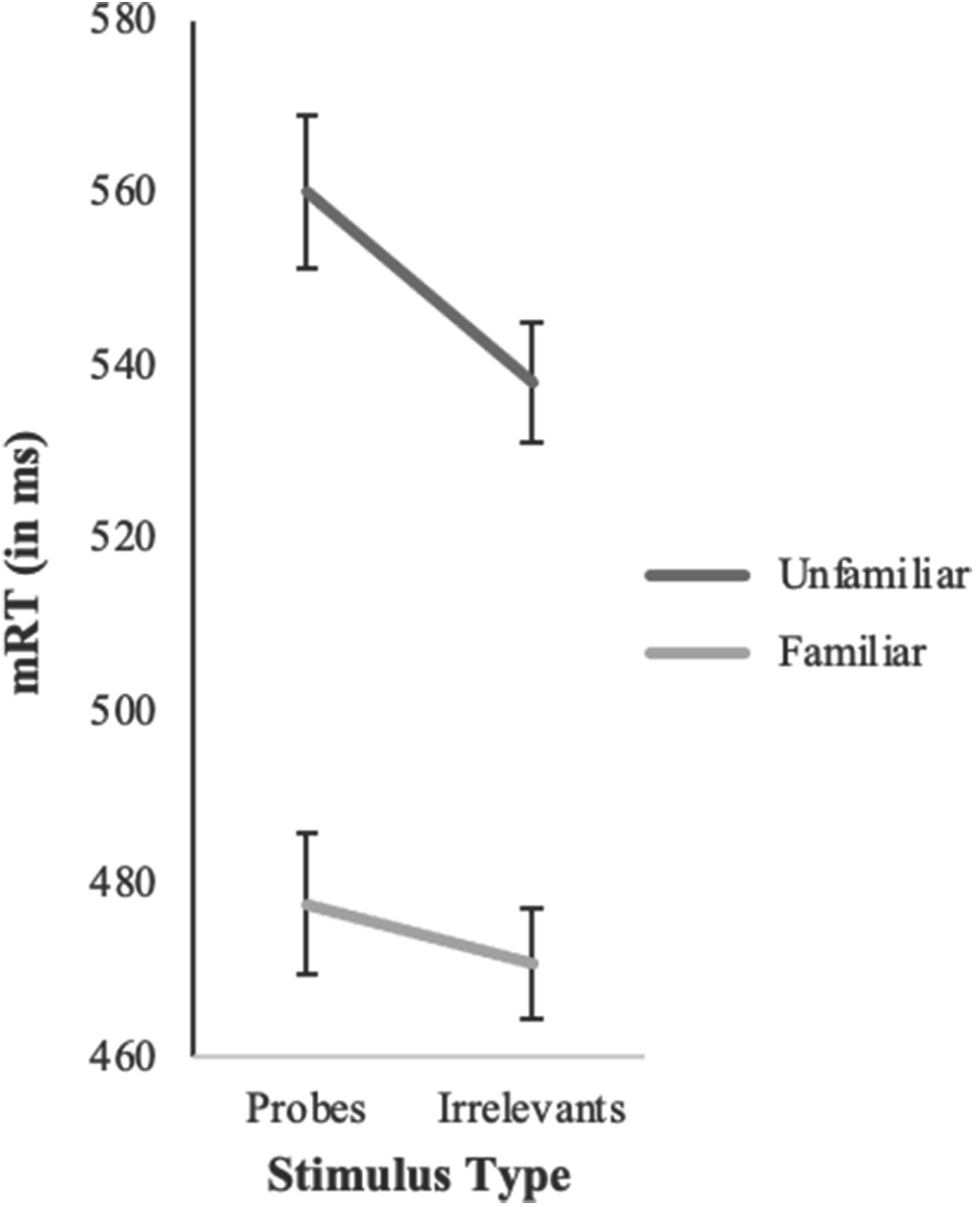
Interaction effect of stimulus type and target-familiarity in Experiment 1. Note: error bars show standard errors

The two main effects were moderated by a significant interaction between stimulus type and target-familiarity, *F*(1,88) = 6.38, *p* = .013, *n*_*p*_^*2*^ = .068. Yet, as Fig. 2 illustrates, the nature of the interaction was opposite to hypothesis 2: The CIT effect was smaller rather than larger for familiar targets compared to unfamiliar targets. For familiar targets, the difference between probes (*M* = 478 ms, *SD* = 54) and irrelevants (*M* = 471 ms, *SD* = 45) was unexpectedly non-significant, *t*(48) = 1.66, *p* = .103, *d* = 0.24, 95% CI [-0.05 0.52]. For unfamiliar targets, reactions to probes were significantly slower than to irrelevants (probes: *M* = 560 ms, *SD* = 61; irrelevants: *M* = 538 ms, *SD* = 43), with a moderate to large effect size, *t*(40) = 4.94, *p* ˂ .001, *d* = 0.77, 95% CI [0.42; 1.12].

To evaluate the strength of the evidence, we also computed a Bayesian mixed measures ANOVA using JASP 0.18.3.0. The model that fit the data best was the model that included the interaction of stimulus type and familiarity and the main effects (BF_M_ = 13.41). The model with interaction fit the data 2.99 times better than the model that included only the main effects (the JASP outputs are available on https://osf.io/j6vuf/?view_only=). This indicates that celebrity targets reduced the CIT effect in Experiment 1.

In Experiment 2, the main effect of stimulus type was again significant, *F*(1,81) = 70.92, *p* < .001, *n*_*p*_^*2*^ = .476. Supporting hypothesis 1, reaction times to probes (*M* = 525 ms, *SD* = 53) were significantly slower than to irrelevants (*M* = 503 ms, *SD* = 49), *d* = 0.95, 95% CI [0.69; 1.21], with a large effect size. Additionally, the main effect of familiarity was significant, *F*(1,81) = 53.33, *p* < .001, *n*_*p*_^*2*^ = .397 Specifically, reaction times were slower in the unfamiliar condition (*M*_*Probe*_ = 554 ms, *SD*_*Probe*_ = 41; *M*_*Irrelevant*_ = 528 ms, *SD*_*Irrelevant*_ = 42) than in the familiar condition (*M*_*Probe*_ = 486 ms, *SD*_*Probe*_ = 42; *M*_*Irrelevant*_ = 469 ms, *SD*_*Irrelevant*_ = 38).

The expected stimulus type x familiarity interaction was non-significant, *F*(1,81) = 3.05, *p* = .085, *n*_*p*_^*2*^ = .036. Similar to Experiment 1 and contrary to hypothesis 2, the CIT effect was not larger in the familiar condition, *d* = 0.79, 95% CI [0.40; 1.16], than in the unfamiliar condition, *d* = 1.09, 95% CI [0.73; 1.44]. This finding was confirmed by an additional, non-pre-registered analysis of the Bayesian ANOVA. The model that fit the data best was the model with the two main effects (BF_M_ = 4.31) and the data were about (BF = 0.90) as likely under the model with the interaction as under the model without the interaction (the outputs are available at https://osf.io/j6vuf/?view_only=).

## Exploratory analyses

### CIT effect for thief and victim

Consistent with earlier studies (Georgiadou et al., 2022; Sauerland et al., 2019) but deviating from the pre-registration, we report the results across probes but also separately for thief and victim. As illustrated in Table 2, the results for the separate analyses for the thief and victim were analogous throughout.

### Error rates

Because error rates are typically very low and lead to less reliable results (Kleinberg & Verschuere, 2015), in line with previous work, we tested our hypotheses exclusively on reaction times. For sake of completeness: in Experiment 1, error rates for probes and irrelevants were very low (*M*_*Probes*_ = 5.80, *SD*_*Probes*_ = 7.98, and *M*_*Irrelevants*_ = 3.16, *SD*_*Irrelevants*_ = 4.80), with higher error rates for targets, *M* = 21.22 (*SD*_*Targets*_ = 10.65). A a 2 (stimulus type: probe vs. irrelevants) x 2 (target familiarity: familiar vs. unfamiliar) ANOVA on the error rates revealed significant main effects of stimulus type, *F*(1,88) = 29.33, *p* < .001, *n*_*p*_^*2*^ = .250, and familiarity, *F*(1,88) = 11.30, *p =* .001, *n*_*p*_^*2*^ = .114. The interaction effect was also significant, *F*(1,88) = 10.66, *p* = .002, *n*_*p*_^*2*^ = .108. Error rates were significantly higher for probes than irrelevants for both unfamiliar, *M*_*Probes*_ = 8.94, *SD*_*Probes*_ = 9.14, and *M*_*Irrelevants*_ = 4.47, *SD*_*Irrelevants*_ = 4.74, and familiar targets, *M*_*Probes*_ = 3.18, *SD*_*Probes*_ = 5.75, and *M*_*Irrelevants*_ = 2.07, *SD*_*Irrelevants*_ = 4.61. In line with the RT findings and contrary to expectations, this CIT effect was again stronger for unfamiliar targets, *t*(40) = 4.63, *p* < .001, *d* = 0.72, 95% CI [0.38; 1.07], than for familiar targets, *t*(48) = 2.27, *p* = .027, *d* = 0.33, 95% CI [0.04; 0.61].

In Experiment 2, error rates for probes and irrelevants were very low (*M*_*Probes*_ = 3.15, *SD*_*Probes*_ = 5.77, and *M*_*Irrelevants*_ = 1.70, *SD*_*Irrelevants*_ = 2.65), with higher error rates for targets, *M* = 15.48 (*SD*_*Targets*_ = 8.51). The 2 × 2 ANOVA on the error rates revealed significant main effects of stimulus type, *F*(1,81) = 5.48, *p* = .022, *n*_*p*_^*2*^ = .063, and familiarity, *F*(1,81) = 6.84, *p* = .011, *n*_*p*_^*2*^ = .078. The interaction effect was non-significant, *F*(1,81) = 2.54, *p* = .115, *n*_*p*_^*2*^ = .030. Error rates were significantly higher for probes than irrelevants for unfamiliar targets (*M*_*Probes*_ = 4.40, *SD*_*Probes*_ = 7.06, and *M*_*Irrelevants*_ = 2.20, *SD*_*Irrelevants*_ = 3.24), *t*(47) = 2.45, *p* = .018, *d* = 0.35, 95% CI [0.06; 0.64], but not for familiar targets, *M*_*Probes*_ = 1.43, *SD*_*Probes*_ = 2.46, and *M*_*Irrelevants*_ = 1.01, *SD*_*Irrelevants*_ = 1.25, *t*(34) = 0.96, *p* = .344, *d* = 0.16, 95% CI [-0.17; 0.49]. This is in line with the RT findings indicating that the CIT effect was stronger for unfamiliar than familiar targets.

### Follow-up photo display

We conducted a nonparametric binominal test against 1/7 odds, with a chance level of 0.14 (Georgiadou et al., 2022). In Experiment 1, participants recognized both the thief (*M* = 0.60 [0.50; 0.70]) and the victim (*M* = 0.64, [0.54; 0.75]) above chance level, *ps* < .001. Recognition accuracy in this task did not differ systematically as a function of target familiarity, with BF_10_ = 1.77 for the thief and BF_10_ = 0.26 for the victim.

Likewise, in Experiment 2, participants recognized the thief (*M* = 0.67, [0.57; 0.78]) and the victim (*M* = 0.70, [0.60; 0.80]) above chance level, *ps* < .001. Recognition accuracy in this task did not systematically differ as a function of target familiarity, with BF_10_ = 0.89 for the thief and BF_10_ = 0.26 for the victim.

## Discussion

In two pre-registered experiments, we examined the effect of target-familiarity on the usefulness of the reaction time-based Concealed Information Test for diagnosing face recognition. In both experiments, reaction times were longer to probes than to irrelevants in all conditions, indicating the expected CIT effect. Effect sizes were moderate to large (Experiment 1: *d* = 0.47; Experiment 2: *d* = 0.95 across conditions). Consistent with previous findings (e.g., Georgiadou et al., 2022; Sauerland et al., 2023b), the RT-CIT was an effective tool for face recognition. Contrary to expectations, however, the familiar targets did not increase the validity of the test. In Experiment 1, celebrity targets unexpectedly even reduced the size of the CIT effect (cf. hypothesis 2). In Experiment 2, the expected interaction between familiarity and CIT effect did not emerge.

We were unable to replicate previous findings on the moderating effect of target-familiarity (Suchotzki et al., 2018). Of note, another recent study failed to replicate the target familiarity effect. Koller et al. (2024) used names, birthdates of friends, and the address of their former residing city as familiar targets. Using familiar targets did not increase the RT-CIT effect. One possible explanation for our findings could be that familiar targets made the task easier, as evidenced by faster reaction times and lower error rates in the familiar vs. unfamiliar conditions in both experiments. Familiar targets do not need to be learned after all (Koller, 2022). Due to the familiarity, focusing on a single feature of the target may have been enough for participants to recognize the celebrities. Consequently, the stimulus may not have been processed during decision making. Reliance on multiple features to increase response conflict, and hence the CIT effect, is known as the target focus hypothesis (Koller et al., 2022).

Another possible explanation for our findings could be that the celebrity targets stood out too much from the other facial stimuli, especially in Experiment 1. Indeed, the familiar celebrity targets in Experiment 1 differed from unfamiliar targets in terms of lighting, amount of make-up, and photo quality and this made them stand out. The RT-CIT effect decreases when targets are too different from the other stimuli. For instance, one study used a joker card as a target while the other stimuli consisted of regular playing cards (Kubo & Nittono, 2008). Reaction times for targets were significantly longer than for the other stimuli and the effect size was moderate in the control condition (*d* = 0.31), but large in the condition participants concealed knowing the probe (*d* = 0.77). Here, the joker might have stood out due to the associated special significance of the card in card games. In our experiments, participants who learnt familiar targets were faster and made significantly fewer mistakes than participants who learnt unfamiliar targets. This was true for probes, targets, and irrelevants. In Suchotzki et al. (2018), however, participants made *more* mistakes in response to probes when targets were familiar to them, compared to unfamiliar. This indicates that our familiar targets condition may have been too easy, reducing the task to a target detection task similar to Kubo and Nittono (2008). Unprompted feedback from participants that the task was rather easy, and the targets were easily distinguishable supports this conclusion.

Evidence that our task may have become a target detection task emerges from the responses to targets in both experiments. Participants responded significantly slower to unfamiliar targets than to familiar targets, with large effect sizes in both experiments (*d*_E1_ = 1.31; *d*_E2_ = 1.24). Furthermore, target error rates were significantly lower for familiar than unfamiliar targets, but only in Experiment 1 (*d*_E1_ = 0.63; *d*_E2_ < 0.01). This suggests that our attempts to decrease the distinctiveness of familiar targets in Experiment 2 was only partially successful. Participants may have detected the familiar targets rather effortlessly, consequently, targets may not have been processed entirely and features that could have increased response conflict did not get incorporated in the decision making process.

Although we were unable to replicate the familiar targets effect, the observed RT-CIT effect sizes are noteworthy in themselves. Consistent with earlier findings (Sauerland et al., 2023a; *d* = 0.65 to 0.87; Sauerland et al., 2023b; *d* = 0.74 and 0.97), we obtained moderate to large CIT effects, with the exception of the familiar targets condition in Experiment 1, where targets arguably stood out the most. The findings from these six experiments combined (the current two experiments; Sauerland et al., 2023a, b) suggest that the RT-CIT may have the potential to for diagnosing facial recognition. This is crucial for the prevention of false identifications.

### Limitations and future directions

This research has several limitations. First, we assumed familiarity based on recognition and knowledge of celebrities’ names and faces using two pilot studies. Knowledge and recognition of a celebrity may however not be sufficient for increasing response conflict. Rather, familiar targets that convey emotional significance and salience to the participants could be imperative for eliciting the expected familiarity effect (Kleinberg & Verschuere, 2015; Suchotzki et al., 2018). To address this issue, future studies may use friends and family members as targets. This may come with privacy concerns and logistical challenges. To protect people’s privacy, researchers would be obliged to obtain consent for several targets per participant, most likely lowering participation rates. Furthermore, participants may not have close ties to people that match the person descriptions of the people in the stimulus events.

A second limitation is that the period between the presentation of the stimulus videos and the administration of the RT-CIT in this study was relatively short. The interval time between the event in question (i.e., the crime) and the CIT in real life would be significantly longer. Detection-accuracy stays relatively strong over time for well-encoded verbal stimuli (Seymour & Fraynt, 2009). Although this has not been tested for facial recognition, this suggests that the current results may generalize to longer retention intervals. Yet, witnesses may not encode perpetrator faces well when events are relatively short. Although observation time did not moderate the CIT effect (Sauerland et al., 2023b), the validity of the RT-CIT may be affected when short observation is combined with a longer retention interval. Longer retention intervals are associated with decreased facial recognition, eyewitness identification (Deffenbacher et al., 2008), and overconfidence in recognition accuracy (Palmer et al., 2013). Future research should investigate whether the RT-CIT is diagnostic of face recognition even with longer time delays between encoding of the event and testing.

## Conclusion

We tested the usefulness of familiar targets as a means to increase the validity of the RT-CIT. While our findings did not support the hypothesis that familiar targets can increase the capacity of the RT-CIT to diagnose face recognition, our results showed moderate to strong capacity to diagnose face recognition in general. These findings add to the accumulating body of research indicating that the RT-CIT can be successful in diagnosing face recognition. Future studies may test whether an emotional significance of target-familiarity or whether other modalities such as target’s names can increase the diagnosticity of the RT-CIT (Koller et al., 2022; Lukács et al., 2017; Suchotzki et al., 2018). An effective RT-CIT could have important implications in the legal field. It could possibly lead to a lower percentage of misidentification due to its reliance on automatic processes and indirect recognition procedure (Sauerland et al., 2019).

## Data Availability

The Inquisit scripts, pilot data, and data are available on the osf: https://osf.io/j6vuf/?view_only=.

## Footnotes

1 Targets are always familiar in the sense that participants memorize them before the start of the CIT. The term ‘familiar targets’ here indicates that the stimuli chosen as targets are known to participants beforehand, beyond the context of the CIT.

2 We used different software to calculate the required sample sizes for Experiment 1 and 2, respectively. Had we used G*Power for Experiment 1, the required sample size would have been 14 participants. Vice versa, had we used MorePower for Experiment 2, the required sample size would have been 208 participants. In hindsight, we have doubts about the MorePower sample size estimates because it deviates strongly from estimates as suggested by G*Power and SPSS.

3 The procedure also included targets as a third stimulus type. Targets, however, are typically not analyzed, as they are not needed to examine the CIT effect.
